# Evaluation of MgO Nanoparticle Foliar Treatment on the Early Stages of Sweet Basil (*Ocimum basilicum* L.) Vegetation

**DOI:** 10.3390/plants15111612

**Published:** 2026-05-24

**Authors:** Dmitry A. Zakharov, Natalia A. Semenova, Eugenia V. Stepanova, Sofia R. Sarimova, Denis V. Yanykin, Sergey A. Shumeyko, Mark O. Paskhin, Ilya V. Baimler, Sergey V. Gudkov, Alexey P. Glinushkin

**Affiliations:** 1 Prokhorov General Physics Institute of the Russian Academy of Sciences, 119991 Moscow, Russia; zaharov121221@mail.ru (D.A.Z.); natalia.86@inbox.ru (N.A.S.); sofyasarimova@gmail.com (S.R.S.); ya-d-ozh@rambler.ru (D.V.Y.); shumik92@gmail.com (S.A.S.); pashin.mark@mail.ru (M.O.P.); ilyabaymler@yandex.ru (I.V.B.); glinale1@mail.ru (A.P.G.); 2 Ishlinsky Institute for Problems in Mechanics of the Russian Academy of Sciences, 119526 Moscow, Russia; 3 Department of Fundamental Sciences, Bauman Moscow State Technical University, 105005 Moscow, Russia

**Keywords:** nanoparticles, MgSO_4_, essential oil, PAM fluorometry, Raman spectroscopy, reactive oxygen species, pigments, gas exchange

## Abstract

Magnesium is essential for photosynthesis and may enhance plant stress tolerance and secondary metabolism, making Mg-based treatments relevant for savory herb production. This study evaluated the effects of foliar application of 5–20 nm MgO nanoparticles (MgO-NPs) at 75, 200, 300, and 600 mg L^−1^; MgSO_4_ at 20 g L^−1^; and aggregated MgO at 300 mg L^−1^ (Bulk) on sweet basil (*Ocimum basilicum* L.) plants grown under total controlled environment conditions. Treatments were applied once at the two-true-leaf stage. MgSO_4_ increased only plant height by 15%, but fresh weight (FW) was not increased. MgO-NPs had no effect on these parameters. However, 600 mg L^−1^ MgO-NPs and bulk MgO treatments reduced FW by 41% and 39%, respectively. Chlorophyll b content increased in all treatment variants, while anthocyanins increased only in variants with 600 mg L^−1^ MgO-NPs and MgSO_4_ treatments. Higher MgO-NP doses induced oxidative stress, reflected by elevated H_2_O_2_ and activation of catalase and ascorbate peroxidase. Bulk caused the highest H_2_O_2_ accumulation and reduced soluble protein content by 26%. MgO-NPs (600 mg L^−1^) increased essential oil concentration by 61%, but not oil yield per plant. High-dose MgO-NPs acted as elicitors of essential oil accumulation, offering a potential strategy for industrial essential oil production despite biomass reduction.

## 1. Introduction

Consumer demand for environmentally friendly plant-derived products has increased in recent decades. Fresh leafy greens are particularly relevant to this market because they provide vitamins, minerals, and bioactive compounds throughout the year. Owing to advances in modern plant cultivation technologies, including the increasing contribution of vertical farming to urban production of leafy greens, leafy vegetables and medicinal plants now represent a substantial share of the vegetable market. Plant extracts containing essential oils are widely studied as alternatives to synthetic compounds because of their antioxidant, antibacterial, antifungal, and other bioactive properties and comparative absence of pronounced adverse effects [1]. In particular, basil plants of the genus *Ocimum* L. have gained considerable popularity for fresh consumption and for the preparation of seasonings, sauces, pastes, teas, essential oils, extracts, and dietary supplements [2]. Leaves of sweet basil (*Ocimum basilicum* L.) are rich in flavonoids and contain, on average, up to 20 essential oil components [3,4], including eugenol and rosmarinic acid, which contribute to antioxidant, anti-inflammatory, and antibacterial properties. The high demand for basil leaves, driven by the expansion of their applications, has made this crop one of the most promising in the global segment of fresh and dried herbs. According to forecasts, the global basil market is expected to grow by USD 397.8 million between 2024 and 2029, with a compound annual growth rate of 4.5% [5]. A major challenge in basil cultivation in vertical farming using LED lighting is the substantially lower accumulation of essential oils, which, depending on the variety, can be ten times lower than under open-ground conditions [6,7]. This may reduce the quality of the harvested fresh yield. Therefore, additional light treatments with ultraviolet radiation and the application of supplementary nutritional or stress-inducing factors are used to improve aroma quality and stimulate essential oil accumulation. Such factors may enhance the production of plant secondary metabolites, including essential oils. A stimulatory effect on oil biosynthesis has been reported for several mineral nutrients, including Mg, Ca, S, and Fe, which have been associated with essential oil biosynthesis and trichome function in aromatic crops of various savory herbs [8]. In crop production and controlled-environment technologies, magnesium is traditionally supplied as a mineral nutrient in the form of readily soluble salts that provide Mg^2+^ to the nutrient solution. In classical nutrient solution formulations, such as modified Hoagland solution, MgSO_4_ is used as a magnesium source [9]. In soil-based agriculture, both fast-acting water-soluble Mg fertilizers, such as MgSO_4_·7H_2_O and Mg(NO_3_)_2_·6H_2_O, and sparingly soluble slow-release sources, such as MgO, Mg(OH)_2_, MgCO_3_, and dolomite CaMg(CO_3_)_2_, are used to correct Mg deficiency. These sources differ in dissolution rate and susceptibility to leaching [10,11]. Magnesium plays a key role in the functioning of the photosynthetic apparatus: Mg^2+^ is the central ion of the chlorophyll molecule and also acts as a cofactor or activator of several photosynthetic enzymes, including RuBisCO. Accordingly, Mg deficiency typically leads to chlorosis and growth inhibition [12]. In addition to its role in photosynthesis, Mg is involved in the redistribution of photoassimilates, enzymatic processes, and antioxidant defense reactions, making it an important element for improving plant productivity and stress tolerance [13]. Since essential oil biosynthesis is part of secondary metabolism and is supported, on the one hand, by photosynthetic products and, on the other hand, can be enhanced by specific stress signals, changes in Mg status and delivery mode may potentially affect not only plant growth and physiological status but also the accumulation and profile of secondary metabolites [13].

The role of Mg in stimulating essential oil production has been investigated in different crops. For example, in peppermint, Mg supplementation in some experiments resulted in a higher essential oil yield and changes in its component composition, including an increase in menthol content and a decrease in menthone content. Shoot elongation and an increase in leaf dry mass were also observed [14]. In other experiments, the total amount of oil per unit mass decreased, but this effect was compensated for by increased yield, expressed as a 25–27% increase in leaf biomass [15].

Magnesium oxide nanoparticles (MgO-NPs) are considered promising agents for stimulating secondary metabolite synthesis because they may induce a moderate oxidative signal through the formation of reactive oxygen species. This signal can activate enzymatic and non-enzymatic mechanisms of antioxidant defense and modulate secondary metabolism [16]. In addition, the nanoscale size of fertilizers can modify the physicochemical properties of the material, including surface area, sorption capacity, and reactivity, which may affect Mg availability and plant physiological responses [17]. However, the efficiency of Mg supplementation depends not only on chemical form and solubility, but also on the physical form of the preparation, namely, ionic, microdispersed, or nanoscale particles, and consequently on the uptake pathway. When using root nutrition, magnesium is absorbed mainly as Mg^2+^; therefore, solid forms such as oxides, carbonates, or metallic Mg must first dissolve and generate ionic forms before Mg^2+^ can be taken up by roots and transported via the xylem [10,12]. When foliar treatments are applied, the situation is different: hydrated ions, such as Mg^2+^ from salt solutions, can penetrate through aqueous or polar pores in the cuticle, whose dimensions impose limitations on the transport of larger particles [18]. In contrast to ions, nanoparticles applied to the leaf surface predominantly enter the leaf through stomata and can subsequently be transported via apoplastic and symplastic pathways [19]. At the cellular level, the cell wall acts as a size-selective barrier. Nanomaterials are more likely to reach the plasma membrane and move within plant tissues when their size approaches the reported cell wall pore diameter of approximately 13 nm [20]. Experimental evidence shows that even for model particles, such as Au nanoparticles of 3–50 nm, particle size and coating chemistry can substantially alter adhesion to the leaf surface, thereby determining the pathway and efficiency of uptake [21]. Therefore, comparing ionic Mg^2+^, nanoscale MgO, and a microdispersed or aggregated Mg form is justified, as these treatments may differ in the rate of available Mg^2+^ formation, uptake pathways, and the intensity of associated stress effects.

Based on these considerations, we hypothesized that the three Mg forms used in the experimental design would induce distinct physiological responses in sweet basil. Mg^2+^ supplied as MgSO_4_ was expected to represent the most readily available ionic form and therefore to induce rapid changes in photosynthetic parameters. MgO-NPs were expected to combine Mg supply with nanoparticle-specific stress or elicitor effects, potentially affecting antioxidant responses and essential oil accumulation in a dose-dependent manner. Aggregated MgO was expected to exert a weaker or more delayed effect because of its lower dispersibility and gradual conversion into plant-available forms.

Although MgSO_4_ is widely used, the comparative effects of nano- and bulk forms of MgO on primary and secondary metabolism in savory herbs remain poorly understood. The aim of this study was to comparatively evaluate the effects of different magnesium forms—MgO-NPs, Mg^2+^ supplied as MgSO_4_, and aggregated MgO (bulk)—on the morphological and physiological parameters of red-leaved sweet basil ‘Zhigolo’ cultivar. Particular attention was given to essential oil accumulation, antioxidant system parameters, and the functioning of the photosynthetic apparatus. This study also assessed the potential application of Raman spectroscopy for monitoring changes in leaf tissue. We previously investigated the effect of magnesium nanoparticles on essential oil accumulation [22]; however, the present work uses a different experimental model, involving a single treatment at the early growth stage, with an emphasis on physiological changes compared with other magnesium forms.

## 2. Results

### 2.1. Characterization of MgO-NPs

The hydrodynamic diameter, ζ-potential, and nanoparticle concentration were determined using a Malvern Zetasizer Ultra particle analyzer (Malvern Panalytical Ltd., Malvern, UK). Measurements were performed in transparent quartz cuvettes measuring 10 × 10 mm, and a Dip Cell ZEN1002 cuvette was used to assess the electrokinetic potential. Nanoparticle morphology and elemental composition were examined by transmission electron microscopy (TEM) using a Libra 200 FE HR microscope (Carl Zeiss, Germany) equipped with energy-dispersive X-ray spectroscopy (EDX). Copper grids were used for TEM sample preparation. Additional assessment of particle morphology was performed by atomic force microscopy (AFM) using an NPX200 microscope (Seiko, Japan).

**Figure 1:**
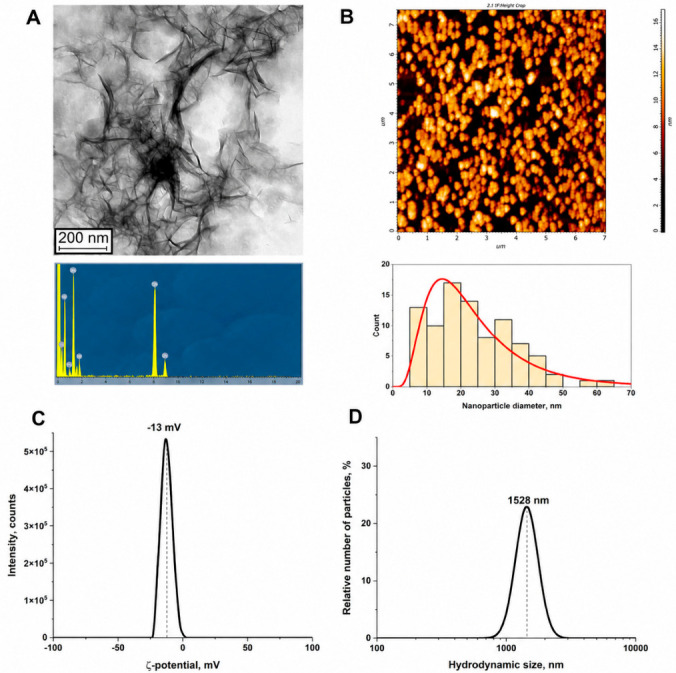
Properties of MgO-NPs synthesized by laser ablation in liquid. (**A**) TEM image of the synthesized MgO-NPs; scale bar, 200 nm; the EDX spectrum of the sample is shown below. (**B**) AFM image of MgO-NPs and particle size distribution histogram based on AFM data. (**C**) ζ-potential distribution of MgO-NPs. (**D**) Number-based particle size distribution by hydrodynamic diameter obtained by dynamic light scattering (DLS) using a particle analyzer.

TEM analysis of the original MgO-NP colloid revealed flake-like MgO nanostructures partially assembled into branched aggregates several hundred nanometers in size (Figure 1A). This aggregation was likely promoted by the use of the undiluted stock suspension (1 mg mL^−1^) and drying on the TEM grid. Because individual particles were difficult to measure reliably from TEM images due to aggregation, the primary particle size was additionally estimated by AFM after dilution and sonication, as described below. The EDX spectrum showed pronounced Mg and O signals, confirming the formation of magnesium oxide.

Particle morphology was additionally characterized by atomic force microscopy (AFM) (Figure 1B). Before analysis, the obtained MgO nanoparticle sample was diluted 200-fold with deionized water and subjected to ultrasonic treatment. AFM imaging revealed individual nanoparticles with a predominantly rounded shape. The particle size distribution of MgO-NPs was reconstructed from the AFM image. According to the size distribution histogram, the majority of particles were in the range of approximately 10–30 nm, whereas the distribution peaked at approximately 5–20 nm. In addition, a small number of larger particles, up to 40–70 nm in size, were present in the sample.

ζ-potential distribution had a maximum at −13 mV (Figure 1C). This indicates the relatively low electrostatic stability of the colloid and a tendency of the particles to aggregate, which is also consistent with the TEM data. In addition, the bulk MgO treatment was characterized by dynamic light scattering and monomodal hydrodynamic size distribution with a maximum at 1528 nm (Figure 1D). The substantially larger hydrodynamic diameter compared with the size of individual particles observed by AFM indicates the presence of aggregates in the aqueous dispersion [22]. Ultrasonic treatment of the MgO-NP colloid at a concentration of 600 mg L^−1^ did not lead to the appearance of flake-like nanoparticle structures for at least 1 h after treatment; therefore, this concentration was selected as the maximum tested concentration. Aggregation processes could have occurred during plant treatment with the nanoparticle suspension or directly on the leaf surface during drying.

### 2.2. Growth and Biomass Parameters

**Figure 2:**
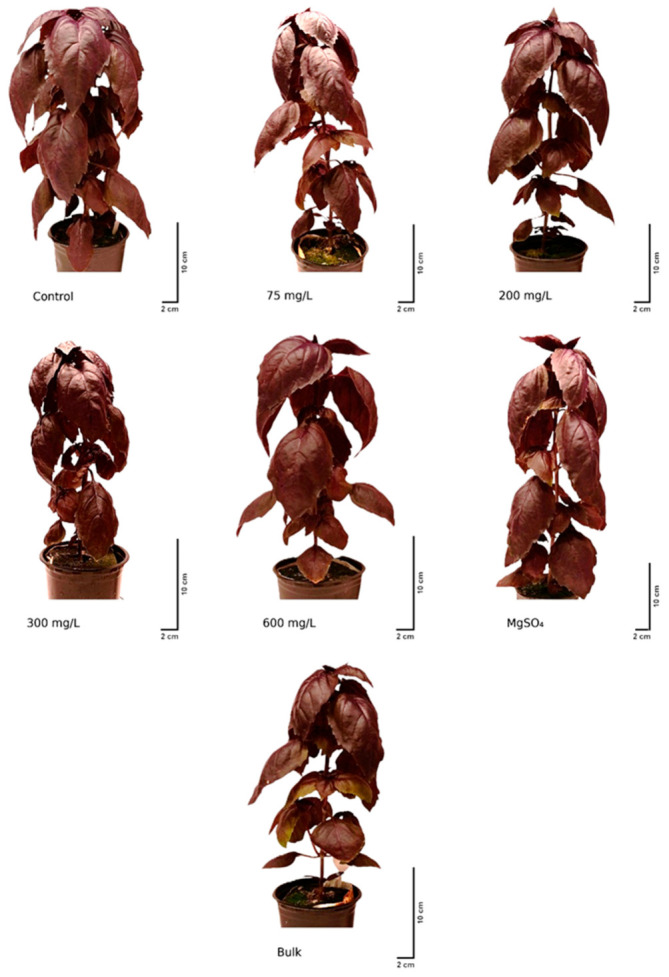
Representative appearance of sweet basil (*Ocimum basilicum* L.) ‘Zhigolo’ cultivar on 50th day after sowing (30th day after foliar magnesium treatment).

**Figure 3:**
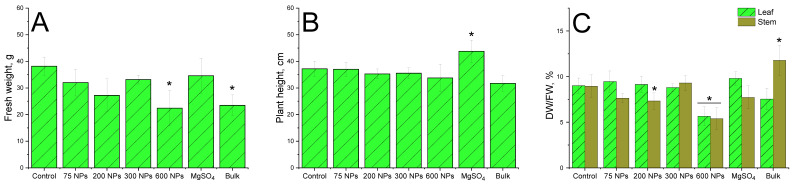
Morphological parameters of red-leaved sweet basil (*Ocimum basilicum* L.) ‘Zhigolo’ cultivar under foliar magnesium treatment: (**A**) fresh weight; (**B**) plant height; (**C**) dry-to-fresh weight ratio, %. All measurements are presented as mean ± SD (n = 10). Asterisks indicate statistically significant differences between treatment and control plants.

On the 50th day after sowing, the growth parameters of sweet basil plants were evaluated (Figure 2). The mean plant height in the 5–20 nm MgO-NPs treatment did not change significantly (Figure 2 and Figure 3B), although a tendency toward reduced height was observed at 600 mg L^−1^. Application of magnesium ions in the form of water-soluble MgSO_4_ resulted in a statistically significant increase in plant height, approximately 15% above the control. The treatment with bulk MgO showed no significant differences in plant height compared with the negative control.

The fresh weight (FW) of control plants was 38 g (Figure 3A). MgO-NP application caused a dose-dependent decrease in FW, from 32 to 22 g within the 75–600 mg L^−1^ concentration range, with the lowest value observed at 600 mg L^−1^, corresponding to a 42.3% decrease. MgSO_4_ treatment did not significantly affect FW compared with the control. In contrast, the Bulk treatment resulted in a pronounced reduction in biomass by 38.4%. Thus, the dose-dependent FW reduction under MgO-NPs indicates growth inhibition rather than a direct nutritional benefit, especially at 600 mg L^−1^.

The dry weight ratio (DW/FW) in the leaves and stems of control plants was similar—approximately 8.9% (Figure 3C). MgO-NP treatments at 75 and 200 mg L^−1^ reduced the DW/FW ratio in stems, while moderately increasing the DW/FW ratio in leaves to 9.4% and 9.1%, respectively. When using the 300 mg L^−1^ treatment, DW/FW values for both leaves and stems returned to the control level, approximately 8.8%. The highest concentration (600 mg L^−1^) caused a noticeable decrease in DW/FW in both leaves and stems to approximately 5.65%, which was about 37% lower than in the control variant plants.

MgSO_4_ significantly increased the DW/FW ratio in leaves by approximately 9% and non-significantly reduced stem DW/FW by approximately 14% relative to the control. In contrast, bulk MgO significantly increased the stem DW/FW ratio to 11.7%, corresponding to an approximately 30% increase, while simultaneously reducing leaf DW/FW to 7.5%, which represented an approximately 16% decrease.

### 2.3. Surface Deposition of MgO Aggregates on Leaves

**Figure 4:**
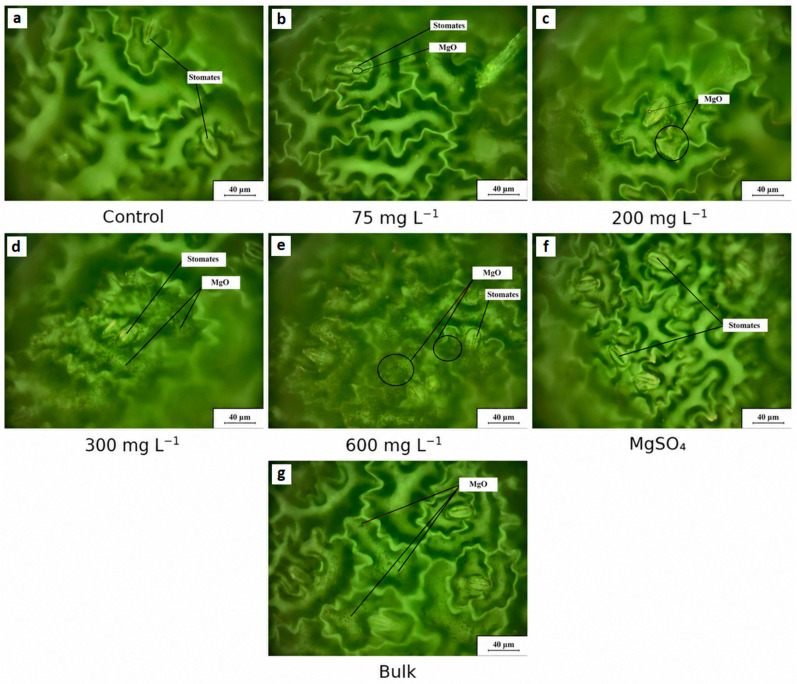
Leaf surface microscopy of sweet basil (*Ocimum basilicum* L.) ‘Zhigolo’ cultivar at ×50 magnification one week after foliar treatments: (**a**) control; (**b**) 75 mg L^−1^ MgO-NPs; (**c**) 200 mg L^−1^ MgO-NPs; (**d**) 300 mg L^−1^ MgO-NPs; (**e**) 600 mg L^−1^ MgO-NPs; (**f**) MgSO_4_ at 20 g L^−1^; (**g**) Bulk MgO suspension.

It is known that the magnitude of the electrokinetic potential depends on the nanoparticle concentration in the colloid [23]: as the nanoparticle concentration increases, the ζ-potential also increases and eventually stabilizes at a certain level. Leaf microscopy after treatments showed the accumulation of particle aggregates of various sizes on the leaf surface. In the MgO-NP treatments, aggregate size ranged from 2 to 4 µm, and their abundance noticeably increased with an increasing concentration from 75 to 600 mg L^−1^ (Figure 4b–e). In the bulk treatment, aggregate size varied from 1 to 10 µm (Figure 4g). There were no visible clusters observed in the control variant and MgSO_4_ treatment (Figure 4a,f).

### 2.4. Pigment Content

Table 1 summarizes the photosynthetic pigment content of sweet basil plants following foliar application of magnesium treatments. Previously, MgO-NP treatment was shown to affect pigment accumulation in leaves [22].

According to Welch’s ANOVA, the treatment effect was statistically significant for chlorophyll *b* content, the Chl a/Chl b ratio, and anthocyanin content, whereas no significant differences among groups were detected for chlorophyll *a*, total chlorophyll, Chl (a + b)/carotenoid ratio, or carotenoid content. MgO-NP treatment increased Chl b content in all variants: at 75–600 mg L^−1^, the increase ranged from approximately 29% to 48% relative to the control, while the greatest increases were observed under MgSO_4_ and Bulk treatments, by 61% and 68%, respectively.

After foliar treatment, the Chl a/Chl b ratio was lower in leaves of all treatment variants than in control plants, indicating a shift toward a relative increase in the concentration of Chl b compared with Chl a. For total chlorophyll, Chl (a + b), statistical analysis revealed no significant differences among groups; however, an increasing trend was observed in the MgSO_4_ and Bulk treatments. Similarly, no statistically significant differences were detected for carotenoid content, although a tendency toward higher values was observed in the MgSO_4_ and Bulk variants. The Chl (a + b)/carotenoid ratio did not change significantly and remained at the control variant level.

For anthocyanin content, the overall treatment effect was statistically significant. Pairwise comparisons with the control showed a significant increase in anthocyanin content only in the 600 mg L^−1^ MgO-NP and MgSO_4_ treatments, by 24% and 30%, respectively, whereas the differences in the 75, 200, and 300 mg L^−1^ MgO-NP treatments and in the Bulk treatment were not significant.

### 2.5. PAM Chlorophyll Fluorescence and Gas Exchange

**Figure 5:**
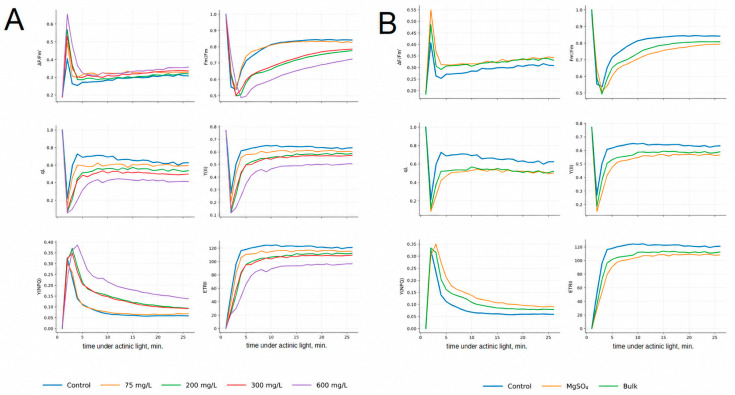
Time-course of chlorophyll fluorescence parameters in sweet basil (*Ocimum basilicum* L.) ‘Zhigolo’ cultivar leaves depending on the treatments: ∆F/Fm′, Fm′/Fm, qL, Y(II), Y(NPQ), and ETR(II). The control variant is indicated by the blue line. (**A**) Treatments with MgO nanoparticles. (**B**) Treatments with MgSO_4_ and Bulk MgO.

PAM fluorometry was used to assess PSII photochemistry and non-photochemical energy dissipation. In the present study, the state of photosystem II (PSII) was assessed to evaluate potential stimulation or inhibition of photochemical processes (Figure 5). After the onset of actinic illumination, the effective quantum yield of PSII (∆F/Fm′) was approximately 0.18–0.20. This parameter is considered to be more sensitive to plant stress than the maximum photochemical efficiency, Fv/Fm. Such values are typical of a physiologically undamaged PSII.

Immediately after illumination, a pronounced response characteristic of the transition from the dark-adapted to the light-adapted state was observed. The effective quantum yield of PSII, Y(II), decreased sharply, reaching a minimum at the second time point in the control. At the same time, the photochemical quenching parameter qL, also decreased, indicating a reduction in the proportion of open reaction centers, whereas the quantum yield of regulated non-photochemical energy dissipation in PSII, Y(NPQ), increased.

Subsequently, during the following minutes after light adaptation, based on the last three measurement points, control plants and plants treated with 75 mg L^−1^ MgO-NPs showed an increase in Y(II) and ETR(II). The qL values also increased and reached steady-state levels, while Y(NPQ) gradually declined to a low background level. This pattern is consistent with normal light-induced acclimation of PSII photochemistry in unstressed leaves.

After treatment with 200–300 mg L^−1^ MgO-NPs and MgSO_4_, a statistically significant decrease in Y(II) and ETR(II) by 9–12% was observed, accompanied by a 22–25% decrease in qL and a 1.7–1.9-fold increase in Y(NPQ) compared with control plants. When using 600 mg L^−1^ MgO-NP treatment, Y(II) and ETR(II) decreased to 77%, qL decreased to 66%, and Y(NPQ) increased approximately 2.8-fold compared with the control variant. In the Bulk treatment, representing aggregated particles, Y(II) and ETR(II) were not different from the control indicators, whereas Y(NPQ) increased significantly by 1.4-fold. Thus, PAM fluorometry data suggest that aggregated MgO does not suppress photosynthetic processes but modifies energy partitioning during photosynthesis.

Previous studies have shown that magnesium application, including magnesium sulfate, can improve the overall state of the photosynthetic apparatus, including under stress conditions, although the effect strongly depends on dose and environmental conditions [24]. However, in the current experiment an opposite pattern was observed, which is more consistent with osmotic effects or contact damage to leaves at high MgO-NP concentrations, leading to a shift in energy distribution toward enhanced photoprotection [25].

**Figure 6:**
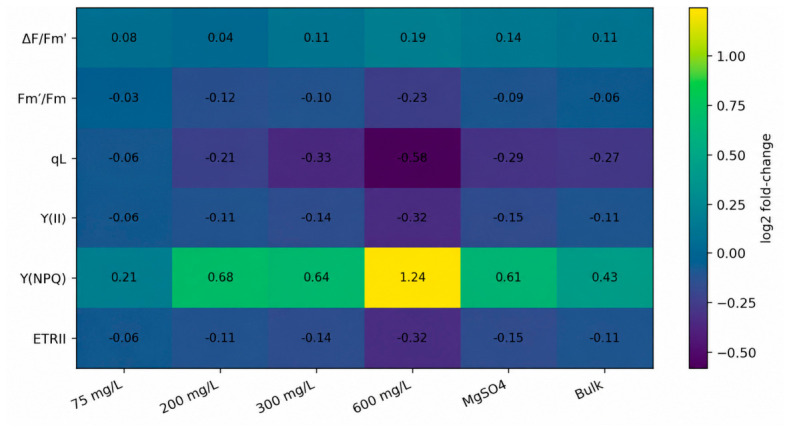
Treatment-induced changes in chlorophyll fluorescence parameters in sweet basil (*Ocimum basilicum* L.) ‘Zhigolo’ cultivar leaves. Heatmap showing log_2_ fold changes relative to the control for ∆F/Fm′, Fm′/Fm, qL, Y(II), Y(NPQ), and ETR(II) under different treatments. Data are means (n = 6).

Figure 6 presents a heatmap of changes in PAM fluorometry parameters relative to the control, expressed as log_2_ fold change. Positive values indicate an increase in fluorescence parameters compared with the control, whereas negative values indicate a decrease. The most pronounced changes were observed following treatment with 600 mg L^−1^ MgO-NPs, where the maximum increase in Y(NPQ) coincided with the most valuable decreases in qL, Y(II), and ETR(II).

In addition to chlorophyll fluorescence, the transpiration rate and CO_2_ assimilation rate were measured. In the dark, at 0 min the CO_2_ assimilation rate (A) was negative in all treatments (Table 2). Compared with the control variant, fewer negative values were observed under 75, 300, and 600 mg L^−1^ MgO-NPs, as well as under MgSO_4_ treatment, whereas no significant differences from the control were detected at 200 mg L^−1^ MgO-NPs or in the Bulk treatment.

Overall, PAM fluorometry showed that low-dose MgO-NPs maintained PSII photochemical performance close to the control, whereas higher MgO-NP doses and MgSO_4_ shifted absorbed light energy from photochemistry toward regulated non-photochemical dissipation, with the strongest response observed at 600 mg L^−1^ MgO-NPs.

Transpiration rate (E) in the dark differed substantially among treatments: the highest values were observed at 75 mg L^−1^ MgO-NPs, whereas the lowest values were recorded at 600 mg L^−1^ MgO-NPs. In the Bulk treatment, transpiration was also significantly higher than in the control, whereas at 200 and 300 mg L^−1^ MgO-NPs and under MgSO_4_ treatment, the values were lower than in the control. Intercellular CO_2_ concentration (Ci) in the dark was higher under 300–600 mg L^−1^ MgO-NPs. Stomatal conductance to water vapor (gH_2_O) in the dark was higher at 75 mg L^−1^ MgO-NPs and in the Bulk treatment than in the control plants. In plants treated with 200 and 600 mg L^−1^ MgO-NPs and MgSO_4_, stomatal conductance was lower than in the control plants.

After the onset of illumination (20 min), CO_2_ assimilation became positive in all treatments; however, the magnitude of light-dependent assimilation strongly depended on the treatment. The most pronounced decrease relative to the control was observed at 200 mg L^−1^ MgO-NPs (by 23.9%). At 75 and 600 mg L^−1^ MgO-NPs, the values were also lower than in the control (by 21.7% and 20.1%, respectively). At 300 mg L^−1^ MgO-NPs, the values remained closer to the control variant but were still statistically reduced (by 6.6%). MgSO_4_ treatment reduced A relative to the control (by 11.8%), whereas the Bulk treatment showed the highest A value (by 8.1% relative to the control).

Under light conditions, the transpiration rate (E) was significantly higher than in the control at 75 mg L^−1^ MgO-NPs (by 21.3%) and 300 mg L^−1^ MgO-NPs (by 5.6%). In the Bulk treatment, absolute values remained close to the control variant. At the same time, transpiration was significantly reduced when using 200 mg L^−1^ MgO-NP treatment and MgSO_4_ treatment (by 30.6% and 23.6%, respectively), and also was below the control level at 600 mg L^−1^ MgO-NP treatment (by 21.6%).

In the light conditions, Ci decreased compared with dark in all treatments. However, differences among treatments remained moderate. The highest values were maintained when using 75 mg L^−1^ MgO-NP treatment (by 5.9%). Higher values were also observed when using 200 and 300 mg L^−1^ MgO-NPs (by 2.5% and 3.0%, respectively). In contrast, 600 mg L^−1^ MgO-NP, MgSO_4_, and Bulk treatment Ci values did not differ significantly from the control.

By contrast, gH_2_O under light conditions showed much stronger treatment-dependent variation. The highest values were observed in the 75 mg L^−1^ MgO-NP (by 70%) variant and in the Bulk treatment (by 50%). At 200 mg L^−1^ MgO-NPs, gH_2_O values were similar to the control ones and did not differ significantly. Stomatal conductance decreased under the MgSO_4_ treatment (by 27.4% relative to the control) declined sharply at 300 mg L^−1^ MgO-NPs (by 46.1%), and reached the lowest values at 600 mg L^−1^ MgO-NPs (by 63.5%).

### 2.6. Oxidative Stress Markers and Antioxidant Enzyme Activities

After foliar magnesium treatments, the redox status of leaves changed mainly due to alterations in hydrogen peroxide (H_2_O_2_) content and the activity of enzymatic components of the antioxidant system (Table 3).

Hydrogen peroxide (H_2_O_2_) levels were lowest under the MgSO_4_ treatment (by 52%). When using the 75 mg L^−1^ MgO-NP treatment, H_2_O_2_ concentration decreased by 30%; 200 mg L^−1^ treatment values did not differ significantly from the control, whereas at 300 and 600 mg L^−1^ they increased by 42% and 27%, respectively. The highest H_2_O_2_ concentration was observed in the Bulk treatment (by 84%). These contrasting responses indicate distinct redox effects of the Mg forms: MgSO_4_ was associated with reduced H_2_O_2_ accumulation, whereas aggregated MgO induced a clear oxidative response. Superoxide dismutase (SOD) activity increased in the 200 mg L^−1^ MgO-NP treatment (by 121%) and decreased in the 75 mg L^−1^ MgO-NP treatment variant (by 68%). The lowest SOD activity was recorded in the MgSO_4_ treatment variant (by 82%). Catalase activity increased significantly mainly under 300–600 mg L^−1^ MgO-NPs (by 37% and 51%, respectively), whereas ascorbate peroxidase (APX) activity showed the strongest increase in the 600 mg L^−1^ treatment (by 86%) and a moderate increase in the 300 mg L^−1^ treatment variant (by 33%). In the Bulk treatment, APX values were not statistically significant but showed an increasing trend (by 28%). APX activity decreased in the MgSO_4_ treatment variant (by 58%). Low-molecular-weight indicators changed less markedly. Ascorbic acid content remained almost stable across all treatments (by 97–100% of the control), whereas free proline content was lower than in the control in all treatments, by approximately 24–60%, with the greatest decrease observed in the Bulk treatment (by 60%). Total protein content decreased significantly under the Bulk treatment (by 26%) and showed a decreasing trend in variants with 75–200 mg L^−1^ MgO-NPs (by 12% and 18%, respectively). In variants with 300–600 mg L^−1^ MgO-NP treatments, total protein remained close to the control level, while it was numerically, but not significantly, higher in the MgSO_4_ treatment (by 6%).

### 2.7. Essential Oil Content

Analysis of essential oil content in red-leaved sweet basil leaves revealed the following results (Table 4).

In the control variant, essential oil content was 0.39% of DW. Essential oil concentration in DW in the 75–300 mg L^−1^ MgO-NP treatment variants did not change significantly, although it showed an increasing trend. The highest concentration, 600 mg L^−1^, resulted in a significant increase in essential oil content (by 61%). In the MgSO_4_ treatment, the values did not differ significantly from the control variant, whereas the Bulk treatment was characterized by the lowest value of this parameter (by 36.5%). When expressed per plant, essential oil yield remained close to the control level in all MgO-NP and MgSO_4_ treatments, whereas Bulk MgO caused a marked decrease from 14.8 to 5.7 µL per plant, corresponding to a 61% reduction.

### 2.8. Raman Leaf Spectroscopy

**Figure 7:**
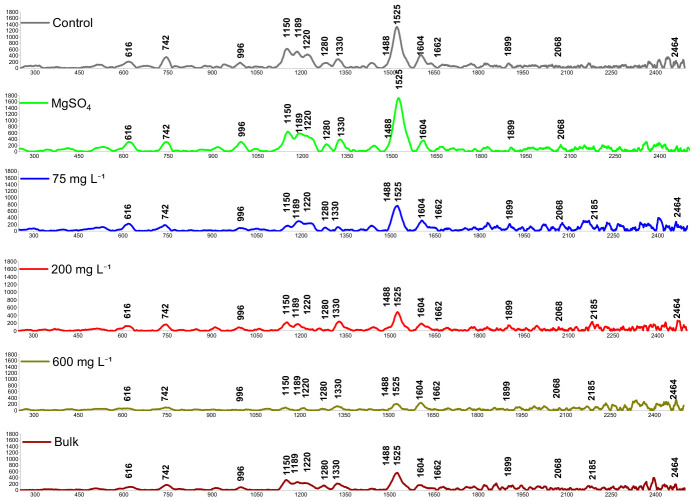
Raman spectra of leaves of sweet basil (*Ocimum basilicum* L.) ‘Zhigolo’ cultivar after foliar treatment with different forms of magnesium, recorded 10 days after spraying. Spectra are shown as means of six spectra per treatment after Savitzky–Golay smoothing.

Raman spectroscopy was used to assess treatment-related spectral changes in sweet basil leaves. When Raman spectra were recorded under 785 nm excitation over the 251–2846 cm^−1^ Raman shift range. The spectra contained characteristic bands associated with leaf structural components and pigments (Figure 7).

When physiological, biochemical, and spectral parameters were compared, the most favorable profile was observed in MgSO_4_-treated plants. In the treatment, the bands associated with chlorophyll at 742, 1280, and 1308 cm^−1^, as well as carotenoid bands at 996 and 1150 cm^−1^, were the most pronounced. This may indicate better preservation or stimulation of the pigment complex compared with the other treatments. Control plants and the 75 mg L^−1^ MgO-NP treatment retained the typical spectrum of a living leaf, with well-defined bands corresponding to chlorophyll, carotenoids, and cell wall components. The treatment with aggregated MgO was close to the 75 mg L^−1^ MgO-NP treatment for most pigment-related bands. The most pronounced changes were observed following treatment with 600 mg L^−1^ MgO-NPs. In the variant, the intensities of bands associated with chlorophyll, carotenoids, and cell wall components decreased, particularly in the regions of 742, 996, 1150, 1280, 1308, and 616 cm^−1^. This may indicate impairment of the photosynthetic apparatus and the development of a stress response. In addition, the 600 mg L^−1^ MgO-NP treatment showed higher signal intensity in the CH stretching region at 2464–2541 cm^−1^. Taken together, the results indicate that leaves of control plants exhibited a typical Raman spectrum of living leaf tissue, with well-defined bands corresponding to the cell wall, chlorophyll, and carotenoids [26]. At the same time, reproducible spectral differences among treatments were observed in specific spectral regions and band shifts.

## 3. Discussion

The comparison among magnesium forms is limited by the non-equivalent Mg concentration in the MgSO_4_ treatment. However, published data on foliar MgSO_4_ application indicate that biologically effective concentrations strongly depend on crop species and application conditions, ranging from low millimolar or ppm levels to percentage-based solutions and several g L^−1^, with clearly defined threshold responses rather than a linear dose effect. For example, the optimum MgSO_4_·7H_2_O concentration was approximately 0.49–0.74 g L^−1^ for grapevine [27], approximately 15 g L^−1^ for damask rose [28], and approximately 20 g L^−1^ for rice grown under saline–alkaline field conditions [29], whereas some other crops experienced pronounced stress already at approximately 0.20 g L^−1^ [30]. Therefore, comparing treatments solely on the basis of nominal mass concentration does not necessarily provide biologically equivalent exposure and may be misleading when MgSO_4_ is compared with MgO-NP-based formulations.

Therefore, the present study should be regarded as a comparison of physiological responses specific to each Mg form, rather than as a strict test of Mg form effects at equivalent Mg concentrations. Future studies should include Mg-equivalent treatments and a broader MgSO_4_ dose range, together with direct measurements of Mg uptake and tissue Mg content, to distinguish nutritional Mg effects from form-specific and stress-related responses. Summarizing the obtained results, the effect of foliar magnesium treatment in sweet basil is determined not only by concentration but also by the form of magnesium, which defines whether the treatment acts primarily as a nutrient source or as a stress-inducing factor. This was particularly evident in growth and biomass traits. MgO-NPs in the range of 75–600 mg L^−1^ did not affect plant height, whereas the soluble ionic form (MgSO_4_), induced a significant increase in height (by 15%). At the same time, MgO-NPs caused a pronounced dose-dependent decrease in FW, with the highest inhibition in the 600 mg L^−1^ variant, whereas MgSO_4_ did not induce visible changes in FW relative to the control. This suggests that MgSO_4_ promotes the formation of more elongated and less compact plants that may reach the maximum shelf height more rapidly, which is undesirable for vertical farming systems. In contrast, MgO-NPs appeared to exert a stronger growth-inhibitory effect than a nutritional one. The DW/FW ratio provides additional insight into these physiological changes. In the control, the dry matter fraction was similar in leaves and stems, approximately 8.9%. At low MgO-NP doses (75–200 mg L^−1^), a tendency toward altered DW/FW distribution was observed, with a moderate increase in leaves and a decrease in stems. This may indicate redistribution of dry matter between organs and potentially reflect early adaptive rearrangements. However, in the 600 mg L^−1^ variant, DW/FW sharply decreased in both leaves and stems. Together with the lowest FW values, this may indicate tissue water accumulation caused by impaired nutritional balance, which is characteristic of a pronounced stress response [31]. MgSO_4_ treatment increased the DW/FW ratio in leaves by approximately 9% without increasing FW, which is consistent with improved leaf tissue quality without an increase in total biomass. In contrast, aggregated MgO reduced FW and caused a contrasting redistribution of the DW/FW ratio between leaves and stems. Quantitative changes in pigment composition support the conclusion that MgSO_4_ acted predominantly as a nutritional treatment, whereas high MgO-NP doses acted as stress-inducing treatments. In all treated variants, Chl b concentration increased and the Chl a/Chl b ratio decreased compared with the control. This pattern suggests an increased relative contribution of light-harvesting complexes (LHCs) and rearrangement of the photosystem antenna system, since Chl b is functionally associated with LHCs [32,33]. From a physiological perspective, this may reflect acclimation involving reorganization of light-harvesting complexes as part of a stress response. The possible relationship with magnesium is not straightforward: Mg^2+^ is an essential structural component of the chlorophyll molecule and affects key processes of the photosynthetic apparatus [34]. However, the selective shift toward Chl b may indicate not merely Mg enrichment, but rather reorganization of pigment–protein complexes, which often occurs when leaf physiological status changes and may accompany stress adaptation [35]. Therefore, the decrease in the Chl a/Chl b ratio in this study should be interpreted primarily as a marker of reorganization of the light-harvesting apparatus rather than as direct evidence of enhanced photosynthesis. Anthocyanin concentration in leaves showed a threshold-type response to the concentration of applied MgO-NPs. A statistically significant increase in anthocyanins was observed at the highest concentration (600 mg L^−1^), and the anthocyanin level in this treatment was approximately comparable to that observed when using the MgSO_4_ treatment. Such an increase also indicates enhancement of antioxidant defense [36].

Functional measurements based on PAM fluorometry and gas exchange are consistent with a regulatory stress-type response of leaves to MgO-NPs. With increasing MgO-NP concentration, Y(II) and ETR(II) decreased, qL declined, and Y(NPQ) increased, which corresponds to the complementary model of quantum yields and to the interpretation of qL as the fraction of open PSII reaction centers [37]. Gas exchange measurements further supported this conclusion. In several treatments, 75, 300, and 600 mg L^−1^ MgO-NPs and MgSO_4_, CO_2_ assimilation decreased, while intercellular CO_2_ concentration increased or remained stable without a proportional decline in stomatal conductance. This may indicate the predominance of non-stomatal limitations, such as limitations in carbon fixation biochemistry or mesophyll conductance. In the 600 mg L^−1^ treatment, this response was accompanied by a pronounced decrease in conductance and transpiration and by the highest photoprotective rearrangement according to PAM data. This suggests reduced electron consumption by carbon metabolism in PSII and a shift toward enhanced photoprotection with restricted photochemistry [38]. The light-induced transition itself, from negative A in the dark to positive A in the light conditions, is consistent with the general physiology of photosynthetic activation [39]. The absence of a strictly uniform dose response, for example, the 300 mg L^−1^ treatment being closer to the control, may also reflect the fact that the effects of foliar Mg application depend on the initial nutritional status and have an optimum, whereas unfavorable doses or application forms may shift the response toward stress [40]. Oxidative stress profiles further support the conclusion that high MgO-NP doses and aggregated MgO forms induce stress. The increase in H_2_O_2_ at 300–600 mg L^−1^, together with increased catalase and ascorbate peroxidase activities, is consistent with a model in which H_2_O_2_ levels and antioxidant enzyme activities reflect the balance between ROS generation and disposal [41]. Notably, the MgSO_4_ treatment showed low H_2_O_2_ levels without pronounced enzyme activation, which is consistent with a healthier physiological state of the leaf and the absence of a strong ROS burden [42]. In contrast, the Bulk treatment was characterized by the highest H_2_O_2_ level and deterioration of protein status, with an insufficient compensatory enzymatic response [43]. This further confirms that magnesium can support photosynthetic and antioxidant characteristics, but its effect depends on dose and application form, and the optimum may shift accordingly [44]. Essential oil concentration in leaves did not change significantly under 75–300 mg L^−1^ MgO-NPs, although a tendency toward an increase was observed. The decrease in essential oil amount per plant was mainly explained by the overall reduction in biomass. The 600 mg L^−1^ MgO-NP treatment caused an increase in essential oil concentration in dry matter, while the amount of essential oil per plant did not increase. This pattern is typical of elicitation, where secondary metabolite synthesis is enhanced under growth-limiting conditions [45]. This elicitor-like effect may involve ROS-mediated stress signaling, as indicated by increased H_2_O_2_ accumulation, activation of CAT and APX, enhanced Y(NPQ), and increased essential oil concentration. However, confirmation of this pathway requires targeted gene expression or transcriptomic analyses. The fact that MgSO_4_ did not increase either essential oil concentration or essential oil amount per plant further supports the conclusion that the form of magnesium application determines differences in the mechanisms of plant response. The decrease in essential oil content under aggregated MgO treatment was likely associated with the unfavorable effects of particle aggregation on the leaf surface, which may reduce treatment efficiency and intensify stress [46].

Raman spectroscopy provided independent spectral evidence supporting this overall interpretation. Living leaves typically show intense carotenoid markers at approximately 1525 and 1150 cm^−1^, as well as bands associated with chlorophyll, including the region around 1604 cm^−1^ and several less intense modes [47]. Against this background, MgSO_4_-treated plants were characterized by the most pronounced pigment-related peaks, which is consistent with the extraction-based pigment data showing increased chlorophyll b and anthocyanin content in this treatment. However, Raman spectroscopy should be interpreted as a complementary spectral method rather than a direct quantitative substitute for pigment extraction [30]. In contrast, treatment with 600 mg L^−1^ MgO-NP concentration resulted in simultaneous suppression of chlorophyll and carotenoid bands, resembling spectral features associated with leaf structural damage [48]. The enhancement of structural contributions, including the CH region and lipid–phenolic components, at high doses is consistent with the observation that, under stress, Raman profiles often shift from pigment-dominated spectra toward stronger contributions from membranes and secondary metabolites [49]. Overall, this dose- and form-dependent pattern is consistent with current concepts of foliar nanoparticle application: at low exposure levels, the effects may be neutral or moderately positive, whereas at higher doses nonspecific stress mechanisms begin to dominate, which is reflected in both spectral and physiological shifts [22].

## 4. Materials and Methods

### 4.1. Plants and Growing Conditions

The sweet basil (*Ocimum basilicum* L.) cultivar ‘Zhigolo’ (Gavrish, Moscow, Russia) was used in this study. The cultivar ‘Zhigolo’ is characterized by a clove/pepper aroma, purple ovate glabrous leaves, inflorescences 15–19 cm in length, and pink flowers. Plant height averages 31–42 cm; the period from full emergence to harvest for fresh herbs is 47–51 days, and the period to flowering is 65–72 days. The yield is 1.0–1.1 kg m^−2^. The cultivar is suitable for cultivation under hydroponic conditions.

Sweet basil plants of the cultivar ‘Zhigolo’ were grown in a phytotron under controlled conditions with fully artificial lighting using SF60BK-1000 LED lamps (Svetolyub, Saint Petersburg, Russia). The photosynthetic photon flux density was 240 ± 10 µmol m^−2^ s^−1^, with a 16 h photoperiod. Day/night temperatures were maintained at 24 ± 1/20 ± 1 °C, and relative air humidity was 55 ± 5%. Mineral wool cubes (Grodan, Wrocław, Poland) were used as the growing substrate. The cubes were placed in irrigation trays and supplied with nutrient solution by the ebb-and-flow method. The nutrient solution was prepared using a three-component hydroponic formulation (Terra Aquatica, Nîmes, France) according to the component ratios recommended for sweet basil (using the declared MgO contents of the fertilizers, the estimated background Mg concentration was approximately 19.5 mg Mg L^−1^).

Foliar treatment of the experimental plants was performed once, until complete leaf wetting, using approximately 15 mL per plant, at the two-true-leaf stage, 20 days after sowing. The following treatments were applied: MgO nanoparticles at concentrations of 75, 200, 300, and 600 mg L^−1^, and Bulk MgO (300 mg L^−1^) used for comparison with the equivalent intermediate concentration at which a significant increase in essential oil content was observed. Treatment with deionized water was used as the negative control, whereas magnesium supplied as MgSO_4_ at a concentration of 20 g L^−1^, corresponding to 4 g L^−1^ Mg, was used as the positive control. In this case, the Mg concentration was not equivalent to the other concentrations tested in the study, because the available literature was insufficient to select an appropriate equivalent total concentration; therefore, we chose a concentration with a previously demonstrated significant positive effect in aromatic and savory herbs [50,51,52,53].

Measurements were performed on the 50th day of cultivation, at the onset of flowering, when essential oil accumulation in the plant reaches its maximum. The following parameters were measured: plant height, fresh weight (FW), dry weight (DW), contents of photosynthetic pigments and anthocyanins, chlorophyll fluorescence and gas exchange, antioxidant system activity, essential oil content, and Raman spectra of leaves.

### 4.2. Synthesis of MgO Nanoparticles

MgO-NPs used in this study were synthesized by laser ablation of a metallic Mg target (99.99%) in a liquid medium. Deionized water with a resistivity of 18 MΩ·cm was used as the liquid medium, with a total volume of 100 mL. The target was placed at the bottom of a 100 mL glass cuvette, with a 2–3 mm layer of liquid above the target surface. An Nd:YAG laser NL300 (Ekspla, Vilnius, Lithuania) was used as the irradiation source with the following parameters: pulse duration, 4 ns; pulse repetition rate, 1 kHz; wavelength, 1064 nm; and pulse energy, 1 mJ. The laser beam was focused on the target surface and moved across it using an LScanH galvanometric optical scanning system (Ateko-TM, Yekaterinburg, Russia) equipped with an F-Theta lens with a focal length of 90 mm. Scanning was performed along linear trajectories within a 2 × 2 cm rectangular area at a speed of 5000 mm/s. The distance between adjacent scanning lines was 30 µm. The total duration of the laser ablation process was 2 h. The resulting stock MgO-NP colloid had a concentration of 1 mg mL^−1^, with a final volume of 100 mL. The particle concentration in the colloid, expressed in mg mL^−1^, was determined by weighing the magnesium target before and after laser ablation using an OHAUS Pioneer PA114C analytical laboratory balance (Parsippany, NJ, USA). Immediately before each treatment, the MgO-NP suspension was sonicated for 30 min in an ultrasonic bath (RS, Corby, UK) to reduce aggregation. An aggregated nanoparticle suspension was used as the bulk treatment, because the aggregate size in this state exceeded 1500 nm; therefore, this suspension was treated as a coarse dispersion.

### 4.3. Pigment Analysis

The contents of photosynthetic pigments were determined after extraction from fresh leaf samples with 100% acetone in six biological replicates, each obtained from an individual plant. For this procedure, fresh second-tier leaves weighing 0.1 g were homogenized and ground in a mortar with acetone, and the extract was then filtered through a glass filter with an appropriate pore size using a vacuum pump. The optical density of the extract was measured with a Cintra 4040 spectrophotometer (GBC Scientific Equipment Pty Ltd., Melbourne, Australia) at wavelengths of 440.5, 662, and 644 nm using a 10 mm cuvette. Pigment concentrations were calculated according to the Holm–Wettstein method for 100% acetone and expressed as mg g^−1^ fresh weight [54]. Final pigment contents were recalculated per fresh weight of the sample. For anthocyanin extraction, 0.3 g of fresh leaf tissue was sampled and similarly ground in a mortar with the addition of 1% HCl. The extract was then placed in a water bath at 44–45 °C and heated for 15 min, after which it was filtered through a paper filter. Anthocyanin content was measured spectrophotometrically at 510 nm. Final values, expressed as mg per 100 g fresh weight, were calculated using the absorption coefficient of cyanidin-3,5-diglycoside in 1% hydrochloric acid solution [55].

### 4.4. PAM Chlorophyll Fluorescence and Gas Exchange Measurements

Chlorophyll fluorescence (FChl), CO_2_ assimilation rate, and transpiration rate in plant leaves were measured using a GFS-3000 gas analyzer integrated with a DUAL-PAM-100 system (Waltz, Eichenring, Effeltrich, Germany). Measurements were performed in a measuring cuvette on intact leaves at room temperature, 25 °C, and 65% relative humidity, under a laminar flow with a CO_2_ concentration of 400 ppm and actinic light at λ = 625 nm and 600 µmol photons m^−2^ s^−1^. Before measurements, plants were dark-adapted for 1 h at room temperature to allow relaxation of light-induced fluorescence quenching, followed by 30 min of adaptation in the cuvette. To determine the effective quantum yield of photosystem II (PSII) in light-adapted leaves, ∆F/Fm′, and FChl parameters after light adaptation, 1–21 min, λ = 625 nm, 600 µmol photons m^−2^ s^−1^, leaves were exposed to a 300 ms saturating pulse, λ = 625 nm, 12,000 µmol photons m^−2^ s^−1^ [56].

### 4.5. Oxidative Stress Markers and Antioxidant Enzyme Assays

The antioxidant status of plants was assessed by quantifying hydrogen peroxide (H_2_O_2_), *L*-ascorbic acid, superoxide dismutase (SOD), free proline, ascorbate peroxidase activity, and catalase (CAT) activity. The contents of H_2_O_2_, *L*-ascorbic acid, free proline, and soluble protein, as well as SOD, CAT, and APX activities, were determined spectrophotometrically using standard methods [57], with a Cintra 4040 spectrophotometer. H_2_O_2_ content was determined according to Bellincampi et al. [58], soluble protein content according to the Bradford method [59], L-ascorbic acid according to Hewitt et al. [60], SOD activity according to Beauchamp and Fridovich [61], CAT activity according to Aebi [62], free proline content according to Bates, Waldren, and Teare [63], and ascorbate peroxidase activity according to Nakano [64].

### 4.6. Essential Oil Content of Sweet Basil

Essential oils (EOs) were extracted with hexane (99.8%). For this purpose, a 2 g sample of fresh third-tier leaves was homogenized and transferred to a flask containing 7 mL of solvent, after which the flask was placed in an ultrasonic bath (RS, Corby, UK) for 15 min. To remove residual moisture, anhydrous sodium sulfate was added to the extract [34], and the extract was then passed through a syringe filter. The total amount of essential oils was determined by gas chromatography with flame ionization detection (GC-FID) using a Chromatec-Crystal 5000 system (Chromatec, Nizhny Novgorod, Russia). An aliquot of the extract, 1 µL, was injected into the gas chromatograph equipped with a BP-1 capillary column, 60 m × 0.25 mm, and Chromatec Analyst software v3.0.0.2. Nitrogen was used as the carrier gas at a constant flow rate of 1 mL/min. Samples were injected in splitless mode. The injector temperature was 200 °C, while the transfer line and detector temperatures were 250 °C. The oven temperature program increased from 70 °C to 220 °C at a rate of 2 °C min^−1^. The total analysis time for each sample was 85 min. Component identification was performed by comparing retention times with those of authentic standards of essential oil compounds and by calculating Kovats retention indices. Quantification was based on the total GC-FID peak area of the detected essential oil components. Values were expressed as essential oil concentration on a dry-weight basis and as essential oil yield per plant. It should be noted that hexane extraction followed by GC-FID characterizes the extractable volatile fraction of basil leaves and may not fully reproduce the essential oil profile obtained by hydrodistillation.

### 4.7. Plant Leaf Raman Spectroscopy

Raman spectra of treated leaves were recorded using a Raman spectrometer (InSpektr, Kazan, Russia). Excitation was performed with a 785 nm laser. The spectral acquisition range was 251–2846 cm^−1^, the spectral resolution was 0.2 nm, the laser power was 1 mW, and the exposure time was 5 s. For each leaf, spectra were acquired at three points, from the apex to the base, after which the mean spectrum was used for analysis. Baseline correction and smoothing using the Savitzky–Golay filter were performed. Peak positions were identified in the processed spectra and subsequently analyzed. The number of biological replicates was three, with n = 6.

### 4.8. Statistical Analysis

For statistical analysis, one-way Welch’s analysis of variance (Welch’s ANOVA) was used. Welch’s ANOVA was used because it does not require the assumption of equal variances among groups. Comparisons were performed between the treatment groups, 75, 200, 300, and 600 mg L^−1^ MgO-NPs, MgSO_4_, and Bulk, and the control variant. For physiological parameters, 10 plants were analyzed in each group across three independent biological replicates (n = 10). All tests were two-sided. Differences were considered statistically significant at *p* < 0.05. When a significant overall treatment effect was detected by Welch’s ANOVA, post hoc comparisons of each treatment with the control were performed using a two-sample Welch’s *t*-test. To control for multiple comparisons, *p*-values were adjusted using the Holm method. For PAM fluorometry, statistical analysis was performed not across all time points simultaneously, but using values calculated as the mean of the last three time points for each plant. Gas exchange parameters measured in the dark, 0 min, and under light, 20 min, were analyzed separately.

## 5. Conclusions

Foliar magnesium treatment of basil showed a pronounced treatment-dependent response. The ionic form (MgSO_4_) acted primarily as an additional nutrient source, improving some growth traits, but it reduced PSII photochemical efficiency and enhanced regulated non-photochemical energy dissipation, and did not enhance essential oil accumulation. By contrast, MgO-NPs influenced as a dose-dependent regulatory factor at low and moderate concentrations, 75–200 mg L^−1^, whereas the high dose (600 mg L^−1^) induced stress-associated elicitation. Aggregated MgO promoted an unfavorable physiological profile and reduced plant productivity, which was accompanied by a decrease in the final essential oil yield and indicates limited efficacy of this Mg form for foliar application. Overall, this study demonstrates that a single foliar treatment with MgO-NPs up to 30 nm in size at a concentration of 600 mg L^−1^ can increase essential oil accumulation in sweet basil plants; however, this effect is accompanied by growth inhibition. This approach may be useful when sweet basil is cultivated for subsequent industrial essential oil production, as it may allow the use of a smaller amount of plant biomass for hydrodistillation within a single production cycle without reducing essential oil yield.

Comparison of our previous studies [3,21] with the present work indicates that a single foliar application, rather than three applications, produces similar dose-dependent patterns of essential oil accumulation. At the same time, additional MgO-NP treatment cycles may further increase essential oil yield, although they are also associated with a more pronounced reduction in plant size. Future studies should focus on dose optimization between 300 and 600 mg L^−1^ MgO-NPs, validation under greenhouse or production-scale conditions, and mechanistic analyses, including tissue Mg determination and transcriptomic profiling of stress- and terpenoid biosynthesis-related pathways.

## Figures and Tables

**Table 1:** Pigment content in sweet basil (*Ocimum basilicum* L.) ‘Zhigolo’ leaves after magnesium treatments.

Group	Control	75 mg L^−1^	200 mg L^−1^	300 mg L^−1^	600 mg L^−1^	MgSO_4_	Bulk
Chl a, mg g^−1^	3.9 ± 0.5	3.8 ± 0.3	3.8 ± 0.3	3.63 ± 0.5	3.6 ± 0.3	4.2 ± 0.4	4.2 ± 0.5
Chl b, mg g^−1^	1.1 ± 0.1	1.5 ± 0.3 *	1.4 ± 0.1 **	1.5 ± 0.4 *	1.6 ± 0.3 *	1.8 ± 0.3 **	1.8 ± 0.4 *
Chl a/b	3.6 ± 0.3	2.5 ± 0.4 ***	2.8 ± 0.2 **	2.4 ± 0.5 **	2.3 ± 0.9 *	2.3 ± 0.4 ***	2.3 ± 0.1 ***
Total chlorophyll, mg g^−1^ FW	5.0 ± 0.6	5.3 ± 0.6	5.2 ± 0.4	5.1 ± 0.8	5.2 ± 0.3	5.9 ± 0.7	6.1 ± 0.8
Carotenoids, mg g^−1^	1.1 ± 0.2	1.1 ± 0.1	1.2 ± 0.1	1.0 ± 0.1	1.1 ± 0.1	1.3 ± 0.2	1.2 ± 0.1
Chl (a + b)/car	4.8 ± 0.7	4.8 ± 0.2	4.6 ± 0.2	5.0 ± 0.4	4.7 ± 0.4	4.6 ± 0.3	4.9 ± 0.2
Anthocyanins, mg 100 g^−1^ FW	33.2 ± 4.7	31.7 ± 4.1	35.8 ± 4.0	33.3 ± 4.9	41.2 ± 3.1 *	43.2 ± 3.3 *	36.0 ± 2.7

Data are presented as mean ± SD (n = 6). Asterisks indicate statistically significant differences relative to the control according to Welch’s *t*-test with Holm correction following Welch’s ANOVA (* *p* < 0.05, ** *p* < 0.01, *** *p* < 0.001).

**Table 2:** Gas exchange parameters measured in leaves of sweet basil (*Ocimum basilicum* L.) ‘Zhigolo’ cultivar. Values are means ± SD (n = 6).

Group	Time	Control	75 mg L^−1^	200 mg L^−1^	300 mg L^−1^	600 mg L^−1^	MgSO_4_	Bulk
A, μmol CO_2_ m^−2^ s^−1^	0 min	−0.74 ± 0.1	−0.62 ± 0.18 *	−0.81 ± 0.06	−0.59 ± 0.05 *	−0.46 ± 0.08 *	−0.55 ± 0.07 *	−0.8 ± 0.08
	20 min	5.79 ± 0.1	4.59 ± 0.14 *	3.88 ± 0.07 *	5.41 ± 0.11 *	4.62 ± 0.09 *	5.1 ± 0.07 *	6.26 ± 0.17 *
E, mmol H_2_O m^−2^ s^−1^	0 min	1.39 ± 0.01	2.28 ± 0.01 *	1.25 ± 0.01 *	1.02 ± 0.01 *	0.51 ± 0.01 *	0.89 ± 0.01 *	1.77 ± 0.02 *
	20 min	2.69 ± 0.09	3.27 ± 0.08 *	1.87 ± 0.02 *	2.84 ± 0.9 *	2.38 ± 0.07 *	2.06 ± 0.13 *	2.66 ± 0.21
Ci, μmol CO_2_ mol^−1^	0 min	304.4 ± 1.1	299.2 ± 2.7	305.7 ± 1.9	310.1 ± 2.1 *	311.5 ± 3.2 *	305.4 ± 1.4	304.9 ± 1.1
	20 min	252.9 ± 4.5	267.7 ± 2.8 *	259.1 ± 4.4 *	260.6 ± 4 *	257.1 ± 3.3	253.3 ± 5.1	256.8 ± 2.9
gH_2_O, mmol H_2_O m^−2^ s^−1^	0 min	221.2 ± 22.5	273.3 ± 24.2 *	172.8 ± 19.3 *	252.4 ± 26.5	194.1 ± 15.8 *	195.4 ± 22.8 *	261.4 ± 18.0 *
	20 min	113.4 ± 11.8	192.4 ± 14.9 *	120.5 ± 14.1	62.3 ± 7.2 *	41.4 ± 3.6 *	82.4 ± 9.7 *	170.4 ± 12.3 *

A, net CO_2_ assimilation rate; E, transpiration rate; Ci, intercellular CO_2_ concentration; gH_2_O, stomatal conductance to water vapor. Asterisks indicate statistically significant differences relative to the control according to Welch’s *t*-test with Holm correction following Welch’s ANOVA (* *p* < 0.05).

**Table 3:** Oxidative stress markers in sweet basil (*Ocimum basilicum* L.) ‘Zhigolo’ cultivar after foliar treatment with different forms of magnesium. Data are presented as mean ± SD (n = 6).

Group	Control	75 mg L^−1^	200 mg L^−1^	300 mg L^−1^	600 mg L^−1^	MgSO_4_	Bulk
H_2_O_2_, µmol g^−1^ FW	2.55 ± 0.46	1.78 ± 0.3 *	2.66 ± 0.29	3.61 ± 0.39 *	3.25 ± 0.96 *	1.215 ± 0.365 *	4.68 ± 0.36 **
AsA, mg g^−1^ FW	0.74 ± 0.01	0.72 ± 0.02	0.73 ± 0.01	0.72 ± 0.018	0.74 ± 0.009	0.741 ± 0.021	0.74 ± 0.007
Pro, µmol g^−1^ FW	0.05 ± 0.02	0.03 ± 0.002 *	0.03 ± 0.01 *	0.038 ± 0.001 *	0.029 ± 0.003 *	0.032 ± 0.002 *	0.03 ± 0.002 *
SOD, U mg^−1^ protein	0.34 ± 0.11	0.11 ± 0.04 *	0.75 ± 0.27 **	0.41 ± 0.17	0.38 ± 0.19	0.06 ± 0.04 **	0.37 ± 0.29
CAT, µmol H_2_O_2_ min^−1^ mg^−1^ protein	23.98 ± 3.21	17.88 ± 2.23	25.25 ± 5.5	32.82 ± 2.16 *	36.24 ± 3.55 *	18.10 ± 5.66	24.19 ± 2.37
APX, µmol AsA min^−1^ mg^−1^ protein	0.36 ± 0.1	0.42 ± 0.06	0.38 ± 0.12	0.48 ± 0.13 *	0.67 ± 0.04 *	0.15 ± 0.104 *	0.46 ± 0.28
Protein content, mg g^−1^ FW	4.96 ± 0.35	4.35 ± 0.1	4.06 ± 0.78	4.99 ± 0.15	4.83 ± 0.38	5.28 ± 0.25	3.68 ± 0.68 *

H_2_O_2_, hydrogen peroxide; AsA, ascorbic acid; Pro, free proline content; SOD, superoxide dismutase activity; CAT, catalase activity; APX, ascorbate peroxidase activity. Asterisks indicate statistically significant differences relative to the control according to Welch’s *t*-test with Holm correction following Welch’s ANOVA (* *p* < 0.05, ** *p* < 0.01).

**Table 4:** Essential oil content and oil yield of sweet basil (*Ocimum basilicum* L.) ‘Zhigolo’ cultivar after foliar Mg treatments. Data are presented as mean ± SD (n = 6).

Treatment	Essential Oil Concentration, % in DW	Essential Oil Yield, µL per Plant
Control	0.389 ± 0.061	14.8 ± 1.2
75 mg L^−1^ MgO NPs	0.440 ± 0.034	14.1 ± 1.1
200 mg L^−1^ MgO NPs	0.466 ± 0.044	14.1 ± 0.9
300 mg L^−1^ MgO NPs	0.451 ± 0.043	14.9 ± 1.2
600 mg L^−1^ MgO NPs	0.626 ± 0.023 **	14.0 ± 1.0
MgSO_4_	0.364 ± 0.070	12.6 ± 2.1
Bulk	0.247 ± 0.056 *	5.7 ± 2.2 *

Asterisks indicate statistically significant differences relative to the control according to Welch’s *t*-test with Holm correction following Welch’s ANOVA (* *p* < 0.05, ** *p* < 0.01).

## Data Availability

The data presented in this study are available on request from the corresponding author (Order of the Director of the Prokhorov General Physics Institute of the Russian Academy of Sciences).
